# Structural variations of pectinate muscles across sheep and rabbit atria

**DOI:** 10.21542/gcsp.2024.15

**Published:** 2024-03-03

**Authors:** Mahmoud A. Sakr, Magdi H. Yacoub

**Affiliations:** 1 Biomedical Engineering and Innovation Laboratory, Department of Research, Aswan Heart Centre, Magdi Yacoub Heart Foundation, Aswan, Egypt; 2 Harefield Heart Science Centre, Magdi Yacoub Institute, Harefield, United Kingdom; 3 Department of Surgery, Aswan Heart Centre, Magdi Yacoub Heart Foundation, Aswan, Egypt; 4 National Heart and Lung Institute, Imperial College, London, United Kingdom

## Abstract

Summary: The venous inflow of each atrial cortex is asymmetric and coupled to geometry and outflow to produce optimal vortices and flow patterns in each chamber. In the right atrium, fiber orientation is dependent on the crista terminals and pectinate muscles, which produce a circumferential squeezing effect to propel blood into the desired direction. The left atrial fiber orientation is a more complex fiber that suits its its geometry and function. This study demonstrates the structural differences between the right and left atria.

Background: The right and left atria play important roles in overall cardiac performance, both at rest and during exercise. Atrial dysfunction due to congenital or acquired heart diseases can result in significant disability or death. The prevalence of such conditions has been rising due to the increasing age of the population as well as the progressively larger number of patients with Grown-up congenital heart disease (GUCH).

Methods: Left and right atria were collected from rabbits and juvenile sheep, and pattern recognition and image analysis were used to illustrate the microstructure and orientation of the pectinate muscles.

Results: The aim of this study is to observe the differences in the structure of the pectinate muscles in both rabbits and sheep. Through image analysis and pattern recognition, we were able to identify the orientation of the patterns that can help produce off-the-shelf patches that are capable of mimicking and/or reproducing most of the functions of normal atrial tissue.

Conclusion: The microstructure of the pectinate muscles is unique and provides remarkable functionality to the atria.

## Introduction

Notable examples of congenital heart disease include those treated with the Fontan procedure. Irreversible damage to atrial tissue requires the availability of sheets of tissue-engineered patches capable of contraction in harmony with other components of the cardiac tissue. The manufacturing of such patches requires a thorough understanding of the biomechanics of the normal atria, the sophisticated flow characteristics in the cavities, and the design characteristics and biological and electrical properties of the atrial wall at the cellular and molecular levels. The current focus of research is to develop a ventricle patch that can support the outer wall of the ventricle and support systole pressure^1^. Optimizing the fabrication process of the patches and choosing the materials that will form the patch is an important step that research groups focus on to develop working patch^2^. In this study we aim to focus on the endocardial wall of the atrium and the structure of the pectinate muscles located in the atrium^3^. One of the current diseases that support the need for a printed atrial patch is mono-ventricle congenital disease^4^. The pectinate muscles (PM) (musculi pectinati) are parallel ridges in the walls of the atria of the heart^5^. They are so-called because of their resemblance to the teeth of a comb, as in pectinate. Behind the crest (crista terminalis) of the right atrium, the internal surface is smooth.

## Materials and methods

### Sample preparation

This study followed the protocol that was approved by the ethics committee. Three rabbits and juvenile sheep atria were examined.

## Results

The pectinate muscles make up part of the wall in front of the right atrial appendage. Atrial infoldings increase the surface area of the atrial chamber at times of dilatation, similar to music instruments. Therefore, these macrofolds (like intestinal villi) help overcome the constantly changing volume status of the right atrium. Since the variation in left atrial blood flow is not significant, the pectinate muscles are not well developed in the left atrium. The same rules explain why there are more trabeculations in the right ventricle than in the left ventricle^6^.

**Figure 1.**
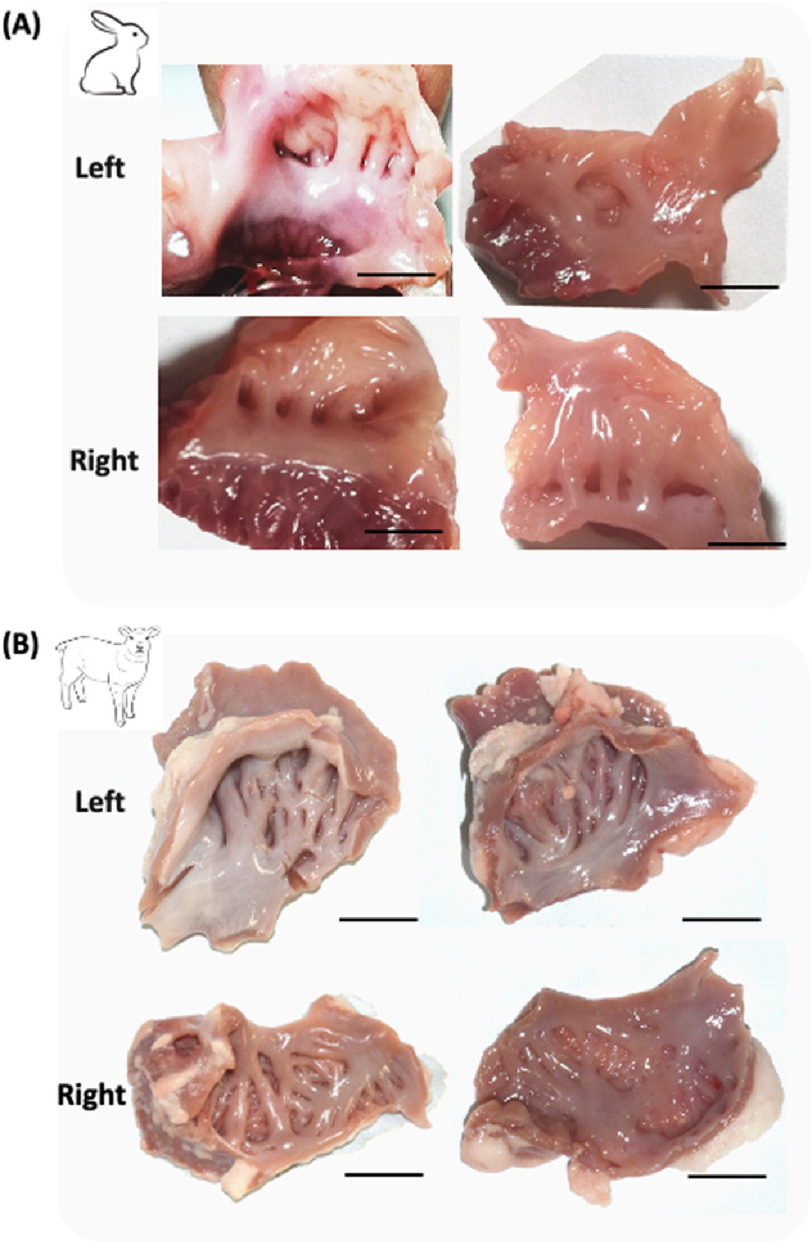
Structure of pectinate muscles. (A) Pectinate muscles from rabbits in both the left and right atria; (B) Pectinate muscles collected from sheep for both the left and right atria. (Scale bar 1 cm).

As shown in Figure 1(A) and (B), the right atrium (RA) is comprised of several components: (1) a vestibule, a smooth muscular rim encircling valvar orifices; (2) a venous component with smooth walls, known to receive superior and inferior caval veins; (3) an appendage, a large triangulated shape positioned anteriorly and laterally, featuring a terminal crest (a fat-filled groove) along its lateral wall that separates its rough wall from the smooth venous sinus, housing the sinus node within this groove; (4) a septum shared between the two atria; and (5) the eustachian valve, triangular in shape and located medially to the Eustachian ridge (the border of the oval fossa and coronary sinus). One of its borders outlines the position of the AV node and its inferior border forms the orifice of the coronary sinus contained in the vestibule, creating the “septal isthmus”. The septal isthmus is often targeted for slow pathway ablation in atrioventricular nodal re-entrant tachycardia, whereas the area of musculature near the triangle’s apex is aimed at fast pathways.

The left atrium (LA) is composed of several features: (1) a venous component, (2) a vestibule encircling the mitral valve, and (3) an appendage, a small finger-like extension with lobes that could potentially serve as a site for thrombus deposition. Despite the absence of a terminal crest in the LA, there is a dividing wall between the rough and smooth walls. Additionally, (4) pulmonary venous components contribute to its structure. The superior wall of the LA is smooth and relatively thicker (measuring 3.5–6.5 mm in formalin-fixed normal specimens), whereas the anterior wall behind the aorta is comparatively thin. The sinus node, which is approximately three mm thick and 10 mm long, poses challenges for ablation from the atrium due to the thickness of the terminal crest. Transitional cells, typical nodal cells, and ordinary myocardium are found at the border of this node. The excitation wave of the sinus node propagates along routes dictated by atrial geometry, given the absence of a specialized link between cardiac nodes. Similarly, the atrioventricular node, arranged as the sinus node, receives input from histologically distinct transitional cells from both atrial walls and the inferior margin of the atrial septum. Internodal conduction is modulated by the organization of myocardial fibers in the intermodal areas.

**Figure 2.**
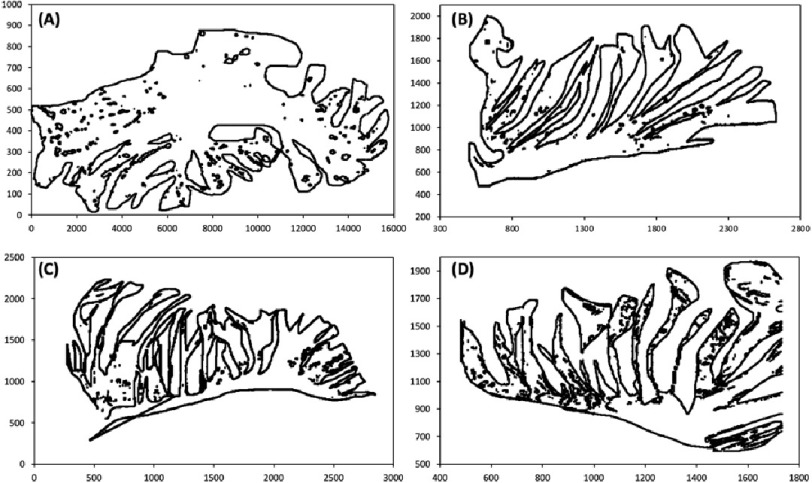
Pectinate muscles pattern recognition. (A) and (B) Pectinate muscles in the right atrium, (C) and (D) Pectinate muscles in the left atrium.

From the images collected from the samples, it was noticed that there was a difference in the structure between the right and left atria in both animal models. As shown in Figure 1(A), the left and right atria of rabbits were identical; however, the left and right atria of sheep were different. This difference was recognized using image analysis and pattern recognition, as shown in Figure 2. This difference is because of the difference in the functionality of both atria. These differences in patterns are represented in terms of the dimension and orientation. These patterns have the potential for 3D printing of cardiac patches for the atrium. Figure 2(A) and (B) represent the thick and coarse pectinate muscles in the right atrium. While Figure 2(C) and (D) demonstrates the thin and smooth muscles which is the case of the left atrium.

## Discussion

The study involved obtaining left and right atrial samples from rabbits and juvenile sheep and employing pattern recognition and image analysis to depict the microstructure and orientation of the pectinate muscles. The primary objective was to examine variations in the structure of these muscles between rabbits and sheep. Utilizing image analysis and pattern recognition, we successfully determined orientation patterns, offering insights that could contribute to the development of readily available patches capable of mimicking or reproducing the functions of normal atrial tissue upon insertion.

### Limitations

The present study was conducted using a restricted set of animal models. Nevertheless, the results obtained were unambiguous and serve as a foundation for comparable investigations. Comparable outcomes were noted in humans, although they were not explicitly presented in this study.

## Conclusion

The analysis of pectinate muscles in this study may hold promise for applications in tissue engineering. This is achieved by comprehending the intricate functioning of atria.
